# High tibial osteotomy achieves unloading of the medial tibiofemoral compartment irrespective of arthritic status or medial collateral ligament release: A biomechanical assessment

**DOI:** 10.1002/jeo2.70633

**Published:** 2026-01-21

**Authors:** Hassan Syed, Aadil Mumith, David Wasserstein, Naveen Chandrashekar, Aaron Nauth, Cari Whyne, Sebastian Tomescu

**Affiliations:** ^1^ Institute for Medical Science University of Toronto Toronto Ontario Canada; ^2^ Department of Surgery, Division of Orthopaedic Surgery University of Toronto Toronto Ontario Canada; ^3^ Holland Bone and Joint Program, Sunnybrook Research Institute Toronto Ontario Canada; ^4^ Basingstoke & North Hampshire Hospital Basingstoke UK; ^5^ University of Central Florida Orlando Florida USA; ^6^ University of Waterloo Waterloo Ontario Canada; ^7^ St. Michael's Hospital Toronto Ontario Canada; ^8^ Department of Surgery, Institute of Biomedical Engineering University of Toronto Toronto Ontario Canada

**Keywords:** medial compartment osteoarthritis, medial open‐wedge high tibial osteotomy, superficial medial collateral ligament release, tibiofemoral contact pressure, varus alignment

## Abstract

**Purpose:**

The purpose of this study was to determine the impact of (1) healthy versus arthritic knees and (2) partial versus complete release of the superficial medial collateral ligament (sMCL) on tibiofemoral compartment pressures at varying mechanical axis alignments following medial open‐wedge high tibial osteotomy (MOW‐HTO).

**Methods:**

Contact pressure (CP) was measured in seven cadaver knees (four healthy; three arthritic) under 1000 N axial load. Tests were conducted at constitutional alignment and MOW‐HTO alignment corrections to 50%, 55%, 60% and 65% of the tibial plateau width (TPW) with the sMCL intact and repeated at each alignment following a partial and complete sMCL release. Linear mixed‐effects models assessed differences in tibiofemoral CP with factors (1) arthritic status and alignment and (2) alignment and sMCL condition. Main effects were assessed for each factor followed by pairwise comparisons when significant.

**Results:**

Medial CP in healthy and arthritic knees did not differ in response to varying alignment corrections (*p* = 0.103). The interaction of alignment and sMCL condition was not significant (*p* = 0.61). With the sMCL intact, altering alignment was found to significantly impact medial CP (*p* = 0.01). Medial CP significantly decreased from constitutional alignment to 55% and 60% of TPW by 19.0% and 13.9%, respectively (*p* = 0.02; *p* = 0.02). Additionally, medial CP at 55% of TPW was found to be 6.6% lower than at 50% of TPW (*p* = 0.046) and 10.4% lower than at 65% TPW (*p* = 0.03). sMCL release did not impact medial CP (*p* = 0.24) but significantly increased lateral CP by 10.8% when completely released (*p* < 0.001).

**Conclusion:**

The presence of medial compartment arthritis may not be a significant factor in the loading biomechanics of cadaver knees secondary to MOW‐HTO corrections. MOW‐HTO correction to 55% of TPW was most effective in decreasing medial CP. sMCL release did not impact medial CP; however, it significantly increased lateral CP when the sMCL was completely released.

**Level of Evidence:**

NA, cadaveric study.

## Graphical abstract

Optimal unloading of the medial compartment with high tibial osteotomy corrections of mechanical axis to 55% of tibial width and overloading of the lateral compartment with complete MCL release.

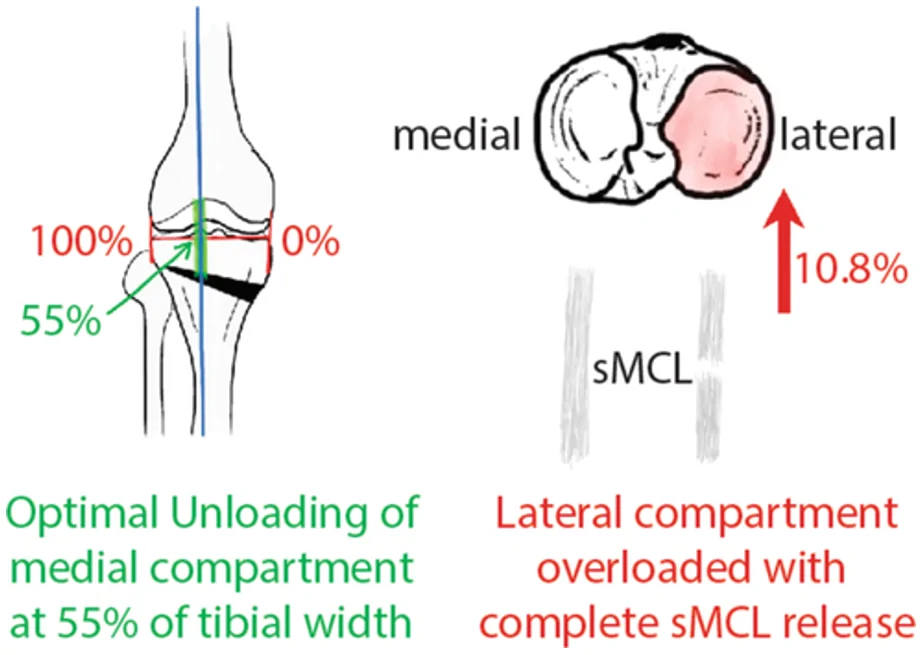

**Abbreviations**
aLDFA: anatomical lateral distal femoral angle
aMPTA: anatomical medial proximal tibial angle
CA: contact area
CP: contact pressure
LCL: lateral collateral ligament
MOW‐HTO: medial opening‐wedge high tibial osteotomy
MTS: materials testing system
OA: osteoarthritis
PCP: peak contact pressure
sMCL: superficial medial collateral ligament
TPW: tibial plateau width

## INTRODUCTION

Medial open‐wedge high tibial osteotomy (MOW‐HTO) surgery is a well‐established treatment option for patients with mild to moderate medial compartment osteoarthritis (OA) and tibial varus deformity [5, 10, 19]. The goal of surgery is to correct proximal tibial varus deformity to shift joint load from the degenerated medial compartment towards the healthy lateral compartment [30]. However, uncertainty remains regarding the optimal post‐operative alignment due to concerns of under‐correction, where original symptoms may persist, and over‐correction, which may lead to lateral compartment OA and patellofemoral issues [6, 9, 11]. Furthermore, the extent to which the superficial medial collateral ligament (sMCL) must be released remains a topic of debate.

Early clinical studies suggested correcting the mechanical axis of the limb to cross the tibial plateau at 62% of the medial (0%) to lateral (100%) tibial plateau width (TPW) [17]. However, correction to 62% of TPW is becoming less common as recent studies have suggested that patients corrected to 50%–55% of TPW have comparable functional and pain scores to patients corrected to the more traditional 62% of TPW [2, 8, 12, 13, 32]. It is thought that patients with less severe arthritis will not require as large of a correction to see improvement in pain and function. Still, some recent research has suggested that the larger corrections have better short‐term functional and regenerative outcomes than smaller corrections highlighting the continued contentious nature of this topic [20, 31]. Additionally, previous in‐vitro biomechanical studies on MOW‐HTO have shown that complete sMCL release may be necessary to effectively unload medial compartment contact pressure (CP) when performing larger corrections to 62% of TPW or a 10° osteotomy [1, 26]. However, the effectiveness of partial and complete sMCL releases has not been investigated across a range of small to large corrections.

In addition, most in‐vitro studies on MOW‐HTO have utilised healthy, non‐arthritic knees which are not representative of the clinical scenario, in which primary indications are medial OA and tibial varus deformity [1, 4, 18, 26]. There are unique intra‐ and extra‐articular morphological differences between arthritic and healthy knees such as cartilage distribution/plateau surface area, joint space loss and bony deformity which are important considerations in the pre‐operative planning of MOW‐HTO [15, 24, 27, 29]. It is unknown if a difference exists in how the medial compartments of healthy and arthritic knees unload in response to MOW‐HTO alignment corrections and sMCL releases. In practice, it is thought that less arthritic knees will require smaller corrections for effective unloading and pain relief; however, this has not been validated from a biomechanical standpoint [14].

The primary aim of this study was to characterise differences in the response of healthy and arthritic knees secondary to MOW‐HTO with respect to CP in the medial and lateral compartments of the knee joint. The secondary aim was to determine the impact of changes in MOW‐HTO alignment and partial and complete sMCL releases in decreasing medial compartment CP.

The primary hypothesis was that MOW‐HTO alignment correction is less effective in unloading the medial compartment of arthritic knees compared to healthy knees. The secondary hypothesis was that a 50% sMCL release is sufficient to reduce medial compartment CP by a minimal clinically significant decrease of 10% at smaller corrections of 50%–55% of TPW, whereas a 100% sMCL release is necessary to reduce mean medial CP for larger corrections of 60%–65% of TPW [16].

## MATERIALS AND METHODS

This experimental cadaveric study investigated the changes in CP in seven cadaver knees (four healthy; three arthritic) under 1000 N axial load. Tests were conducted at constitutional alignment and MOW‐HTO alignment corrections to 50%, 55%, 60% and 65% of the TPW with the sMCL intact, and repeated at each alignment following a partial and complete sMCL release. This represents a total of 15 testing conditions for each knee. The study was approved by the Research Ethics Board at Sunnybrook Research Institute (#1621).

### Specimen preparation and osteotomy

**Figure 1:**
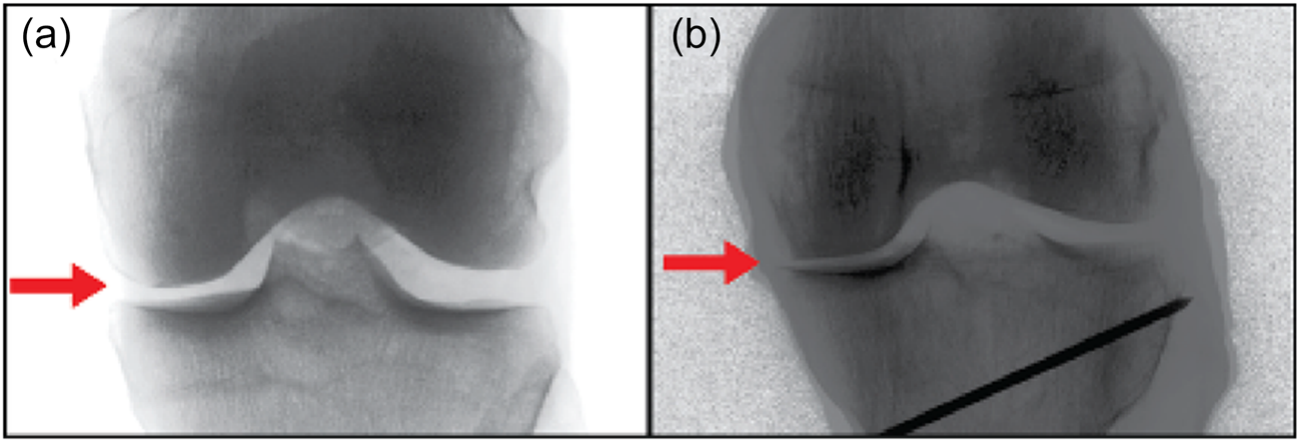
Antero‐posterior radiographic comparison of healthy (a) and arthritic (b) knee. Arthritic knee (b) has medial compartment joint space loss (Kellgren*–*Lawrence grade 2).

Seven fresh‐frozen human cadaver knees were utilised in this experimental cadaveric study. Joints were examined radiographically and visually to categorise into healthy (*n* = 4, mean age: 53 years, range: 43–59 years, two male) and arthritic (*n* = 3, mean age: 59, range: 53–64, two male) groups based on joint space loss and cartilage degeneration on the Kellgren–Lawrence scale using antero‐posterior non‐weightbearing views (Figure 1; Table 1) [7]. The extent of osseous deformity in the distal femur and proximal tibia was assessed by radiographic measurement of the anatomical medial proximal tibial angle (aMPTA) and anatomical lateral distal femoral angle (aLDFA) (Table 1). All arthritic knees had predominantly medial compartment disease.

**Table 1:** Specimen characteristics.

	Knee condition	
	Healthy	Arthritic
*N* =	4	3
Age (mean, range)	53 (43–59)	59 (53–64)
Sex	2 male, 2 female	2 male, 1 female
Kellgren–Lawrence grade	0	1–2
Anatomical medial proximal tibial angle (°)	87.1 ± 2.0	87.5 ± 0.5
Anatomical lateral distal femoral angle (°)	81.6° ± 1.0	82.0 ± 1.9
Constitutional alignment (% of tibial plateau width)	44% ± 5%	37% ± 6%

*Note*: Sample size, mean age (range), Kellgren*–*Lawrence grade, anatomical medial proximal tibial angle (mean ± SD), anatomical lateral distal femoral angle (mean ± SD), and constitutional alignment based on crossing of mechanical axis along tibial plateau as a percentage of tibial width (medial edge = 0%; lateral edge = 100%).

Specimens were thawed over 24 h at room temperature. Each knee was dissected by removing skin, muscles, subcutaneous tissue and extensor mechanism, while leaving cruciate and collateral ligaments, posterior joint capsule, popliteus, menisci and intermeniscal ligaments intact. The diaphyses of the femur and tibia were cut perpendicular to the shaft measuring 15 cm from the joint line. The fibula was cut 6 cm long and fixed to the tibia with a tricortical screw to maintain natural tension of the lateral collateral ligament (LCL).

**Figure 2:**
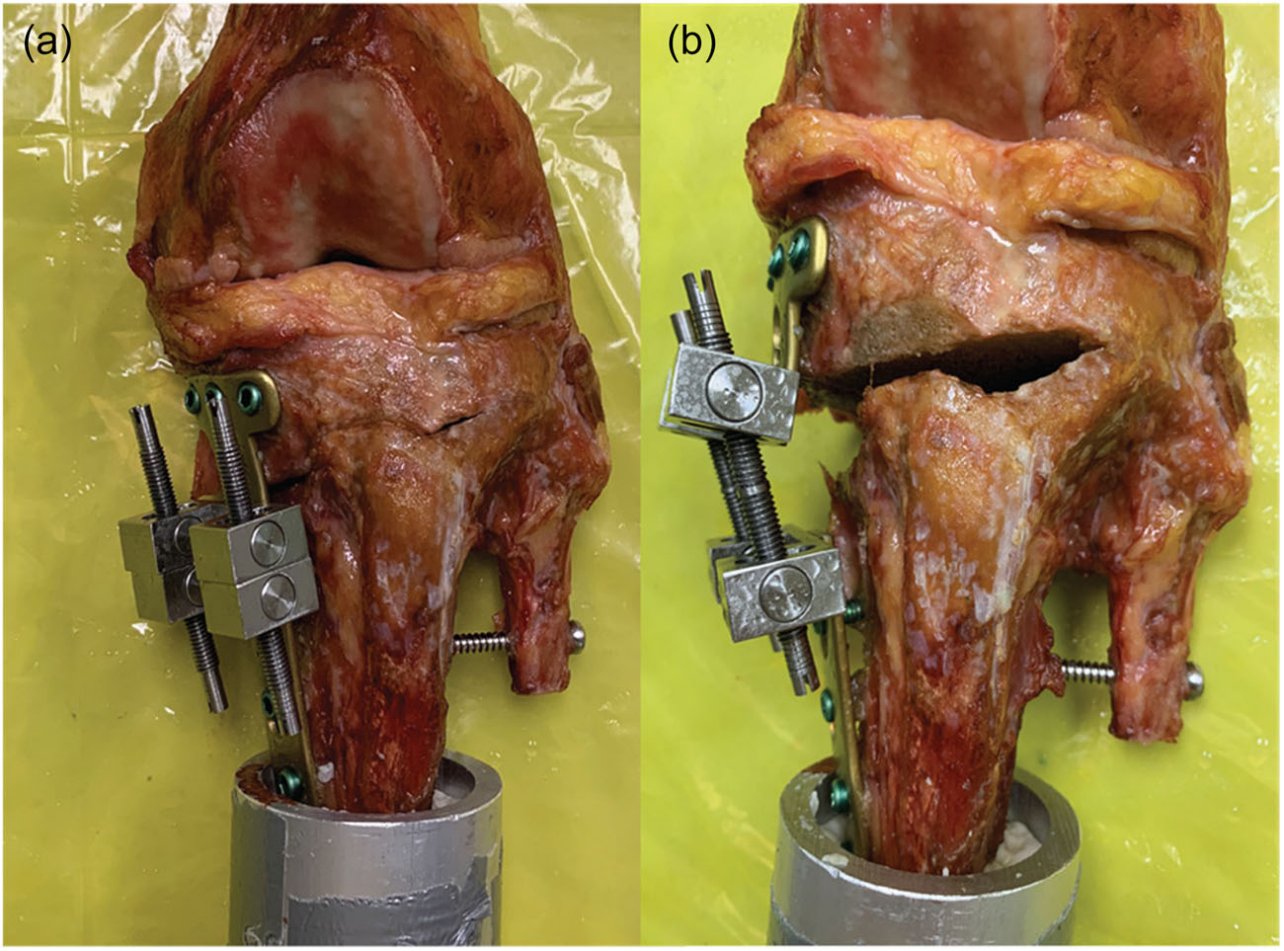
Customised tomofix plate capable of allowing stepless adjustment of osteotomy size. (a) Plate is applied in the closed osteotomy state, where the plate maintains is original shape. (b) Unwinding the double‐sided bolts allows the plate to expand and angulate to accommodate the hinged opening of the osteotomy along an arc.

A fellowship‐trained orthopaedic surgeon performed biplanar MOW‐HTOs according to the Staubli method, leaving 5–10 mm lateral cortex hinges, where hinge position and osteotomy trajectory were controlled and confirmed using fluoroscopy [28]. Without opening the wedge, the osteotomy was fixed with a Tomofix plate (DePuy Synthes) that was customised to allow for stepless adjustment of the wedge size (Figure 2). Two inches of the proximal femur and distal tibia were potted with polymethylmethacrylate bone cement.

### Pressure sensors

Small incisions of the anterior and posterior joint capsule were made just large enough to allow for submeniscal insertion of pressure‐sensitive sensors into the medial and lateral compartments (K‐Scan 4000, 1500 psi; Tekscan Inc.). Teflon tape was applied to the sensors to prevent damage from sharp folding and wrinkling. After optimising the sensors coverage of the tibiofemoral compartments, they were secured by suturing the non‐sensing tabs to surrounding soft tissue structures. Sensors were pre‐conditioned by loading the knee to testing loads (1000 N) three times. Calibration was completed within the knee joint using single point calibration (1000 N) according to the manufacturer's recommendations using a Materials Testing System (MTS; Bionix 858; MTS Systems). CP (MPa) was recorded in the medial and lateral compartments at 60 frames per second throughout the loading cycle.

### Testing apparatus

**Figure 3:**
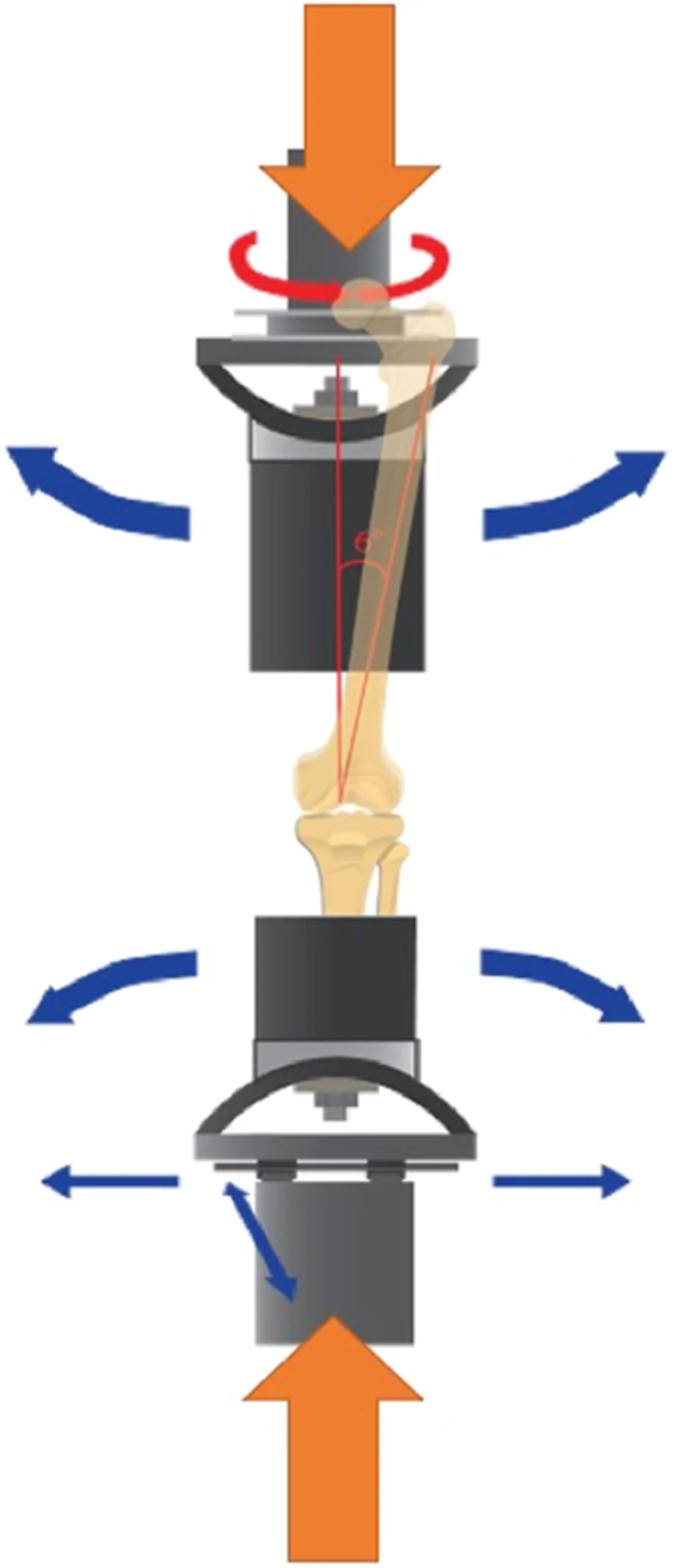
Schematic representation of testing apparatus. Femur and tibia are secured in pots that can slide along convex surfaces to adjust varus‐valgus alignment. Tibial base is capable of translating horizontally in the frontal and sagittal planes. Femoral base is capable of rotating axially. Femur and tibia are potted in a manner that ensures the simulated femoral head and talus remain in line with the loading axis of the materials testing system.

**Figure 4:**
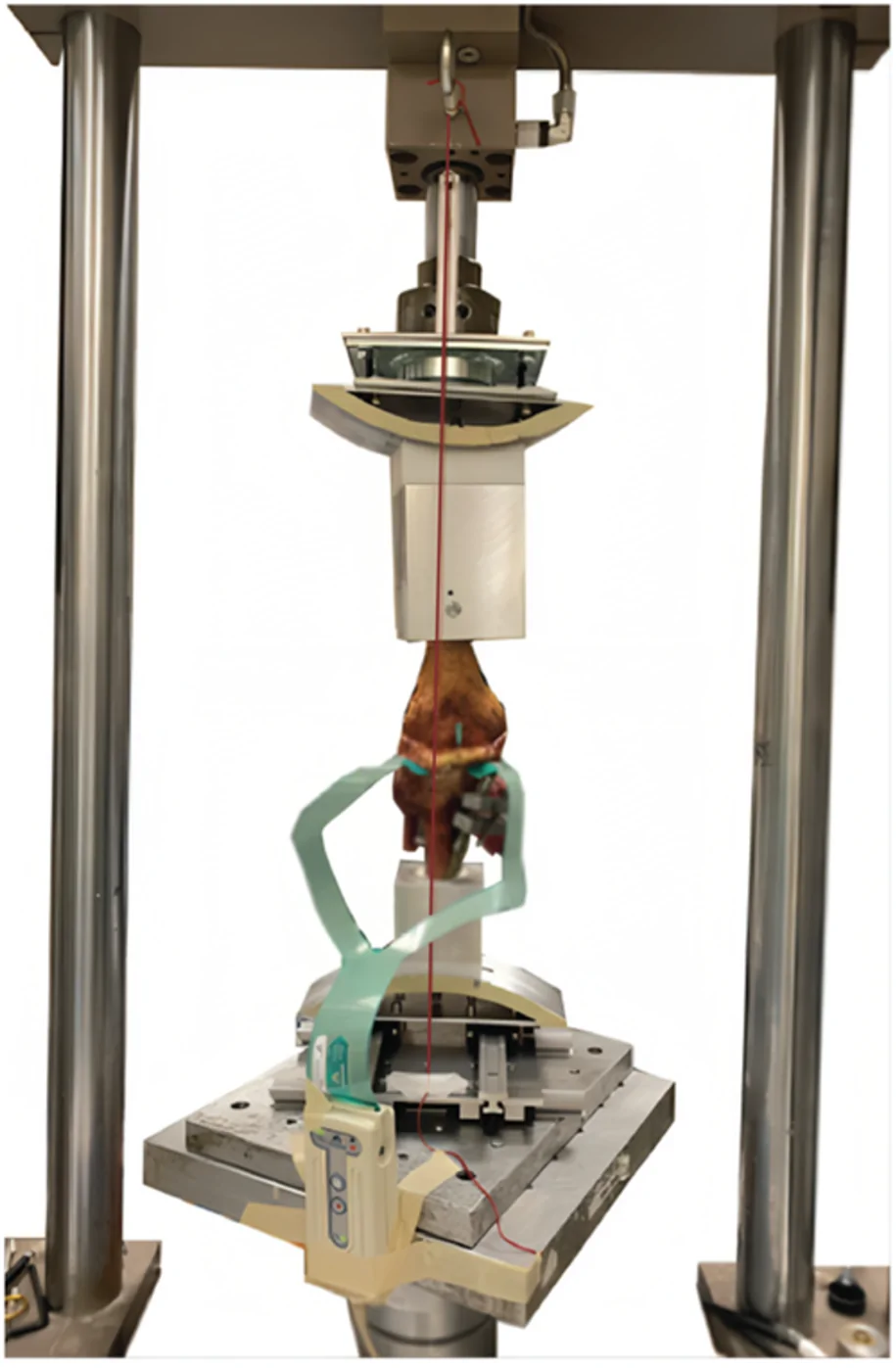
Cadaver knee loaded into testing apparatus. Vertical red string represents the mechanical axis which is used to measure alignment based on percentage of tibial plateau width. Tekscan sensors are inserted submeniscally into the medial and lateral tibiofemoral compartments.

The femur and tibia were loaded onto a custom‐designed testing apparatus that simulated the anatomy of the whole leg in full extension (Figures 3 and 4). This was achieved by potting the shaft of the femur at a 6° valgus to mimic the anatomical femoral axis [21]. Adjustments to alignment in the frontal plane (i.e. varus‐valgus) could be made by sliding the femoral and tibial pots along the convex surfaces. The convex surfaces ensured that the simulated femoral head and tibiotalar joint remained fixed in real space regardless of frontal plane alignment. This allowed the mechanical axis of the limb to remain in line with the fixed vertical loading axis of the MTS and, thus, prevent unaccounted vector forces from being transmitted through the knee. The base of the tibial attachment was capable of free horizontal translation in the frontal and sagittal planes. The femoral attachment was capable of freely rotating around the vertical axis. The free movement allowed the joint surfaces to optimise contact during loading. The entire testing apparatus was fixed to the MTS which was used to apply axial compressive loading.

### Testing protocol

While leaving all movement axes unconstrained, a pre‐load of 250 N was applied to allow the joint surfaces to optimise contact under load. Although true constitutional alignment could not be determined using cadaver knees, the standardised pre‐load setup determined the natural resting alignment and was considered as constitutional alignment for this study. At constitutional alignment, all knees had mechanical axes that crossed the tibial plateau <50% of medial to lateral TPW (Table 1).

The alignments tested included constitutional alignment and post‐operative mechanical axes that crossed the tibial plateau at 50%, 55%, 60% and 65% of the medial to lateral TPW. Measurement of alignment was achieved prior to loading by fixing a string from the simulated femoral head to the tibiotalar joint and using a caliper to measure the crossing point of the string across the tibial plateau. Two separate observers measured alignment twice each to ensure inter‐ and intra‐observer reliability proceeding when measurements were within 2 mm of each other and ±2 mm from desired alignment. The size of the wedge was adjusted/opened by expanding the custom Tomofix plate to achieve target alignments (Figure 2).

**Figure 5:**
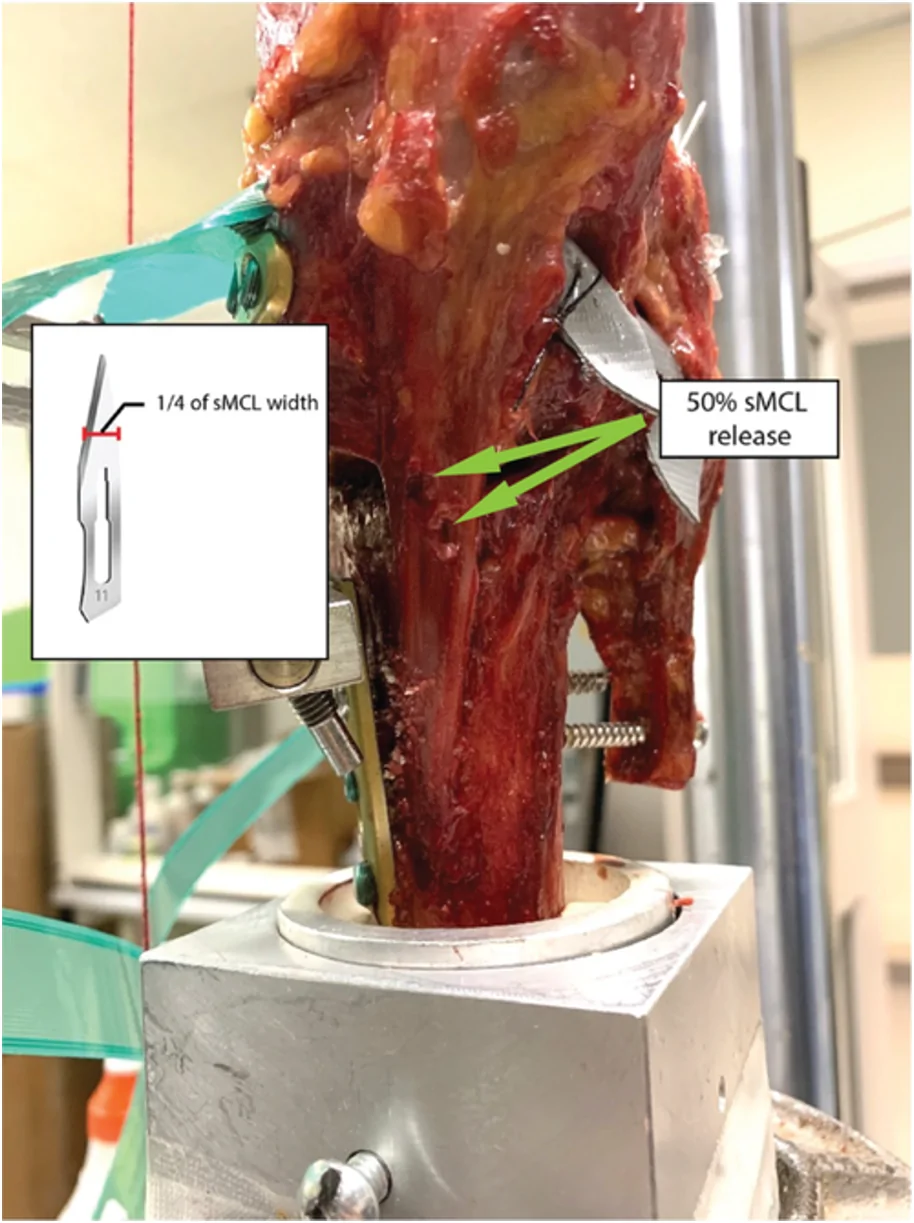
50% release of superficial medial collateral ligament (sMCL) made in a pie‐crusting fashion. A #11 scalpel blade was used to make two staggered incisions at the level of the osteotomy that each measured one‐fourth of the total sMCL width. The 100% sMCL release was carried out by completing the cut horizontally at the level of the osteotomy.

Two trials were conducted for each alignment. The alignment conditions were first tested in sequence from constitutional alignment to 65% of TPW with the sMCL intact. A 50% release of the sMCL was carried out by making two staggered incisions with an 11‐blade scalpel at the level of the osteotomy, each accounting for one quarter of the width of the sMCL (Figure 5). The alignment conditions with the sMCL 50% released were tested in reverse from 65% of TPW to constitutional alignment. Finally, the sMCL was completely released and the alignment conditions were tested from constitutional alignment to 65% of TPW. Randomisation was not possible due to sequential nature of sMCL release and to minimise errors in setting alignment.

Each trial involved loading the knees to a maximum of 1000 N (approximately 1.5 times bodyweight) using a displacement ramp of 4 mm/minute to be consistent with literature [1, 26]. Maximum load was held constant for 10 s and values were collected at the end of the 10 s to account for stress‐relaxation effect. Horizontal translation in the frontal and sagittal planes and the varus‐valgus alignment were locked in place for each trial to avoid unintended changes in alignment during loading; however, rotation in the longitudinal axis was left unconstrained to allow the femoral condyles to optimise contact with the tibial plateau.

### Statistical analyses

Data from similar studies were used in this study design to calculate a priori power to ensure the recruited sample size of cadaver knees was sufficient, with data from both trials used to improve statistical power (GLIMMPSE; α=0.05, n = 3 arthritic knees and n = 4 healthy knees or 70 total observations, SD = 0.1) [1, 25]. The a priori power obtained was >0.8 for the primary hypothesis assessing the interaction effect of arthritic status and alignment on normalised medial CP.

Normality was assessed using a Shapiro–Wilk test revealing approximately normal distribution. Statistical significance was considered when *p* < 0.05.

Linear mixed‐effects models were fitted using lme4 package in R (v1.1.36, Bates et al.). The first model tested for any interaction effect between the factor's arthritic status and alignment followed by models testing the main effect of each factor. The second model tested for any interaction effect between the factor's alignment and sMCL condition followed by models testing the main effect of each factor. Pairwise comparisons were run for statistically significant main effects. CP was normalised by dividing raw pressure values by the pressure in constitutional alignment within each sMCL condition. This allowed for better assessment of proportional changes in pressure from the baseline constitutional alignment and normalised for any differences found when resetting to constitutional alignment between conditions.

## RESULTS

### Specimens

There were four healthy knees (mean age: 53 years, range: 43–59 years, two male; Kellgren–Lawrence grade 0) with an average aMPTA of 87.1° ± 2.0°, aLDFA of 81.6° ± 1.0°, and constitutional alignment of mechanical axis crossing 44% ± 5% of TPW (Table 1). There were three arthritic knees (mean age: 59, range: 53–64, two male; Kellgren–Lawrence grade 1–2) with an average aMPTA of 87.5° ± 0.5°, aLDFA of 82.0° ± 1.9°, and constitutional alignment of mechanical axis crossing 37% ± 6% of TPW (Table 1). All arthritic knees had predominantly medial compartment arthritis.

### Effects of arthritic status

**Figure 6:**
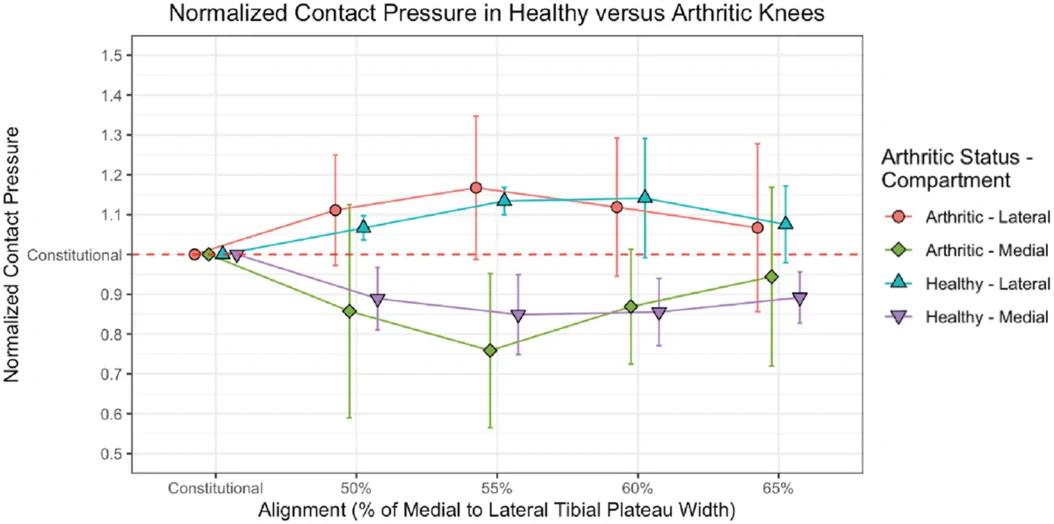
Medial and lateral contact pressure (normalised to constitutional = 1; SD) according to arthritic status and alignment. Medial contact pressure decreased significantly from constitutional alignment to 55% and 60% tibial plateau width (TPW) and was lower at 55% TPW than at 50% and 65% TPW. Lateral contact pressure increased significantly from constitutional to 55% TPW and was higher at 55% TPW than at 50% and 65% TPW.

In evaluating if MOW‐HTO alignment correction is less effective in unloading the medial compartment of arthritic versus healthy knees, the interaction of arthritic status and alignment was not significant for medial or lateral compartment normalised CP (*p* = 0.103; *p* = 0.424)(Figure 6; Table 2). The main effect of arthritic status was also not statistically significant for medial or lateral compartment normalised CP (*p* = 0.93; *p* = 0.73)(Figure 6; Table 2).

**Table 2:** Medial and lateral compartment normalised contact pressure according to alignment and arthritic status.

Knee condition	Alignment (% of medial to lateral tibial plateau width [TPW])	Medial contact pressure (CP) (Mean ± SD; normalised to constitutional alignment = 1)	Lateral CP (mean ± SD; normalised to constitutional alignment = 1)
Healthy (*n* = 4)	50%	0.89 ± 0.08	1.07 ± 0.03
	55%	0.85 ± 0.10	1.13 ± 0.03
	60%	0.86 ± 0.08	1.14 ± 0.15
	65%	0.89 ± 0.06	1.08 ± 0.1
Arthritic (*n* = 3)	50%	0.86 ± 0.27	1.11 ± 0.14
	55%	0.76 ± 0.19	1.17 ± 0.18
	60%	0.87 ± 0.14	1.12 ± 0.17
	65%	0.94 ± 0.22	1.07 ± 0.21

*Note*: Medial and lateral compartment contact pressure (normalised to constitutional alignment = 1) according to arthritic status and alignment in the superficial medial collateral ligament intact condition. Values represent means with standard deviation.

**Figure 7:**
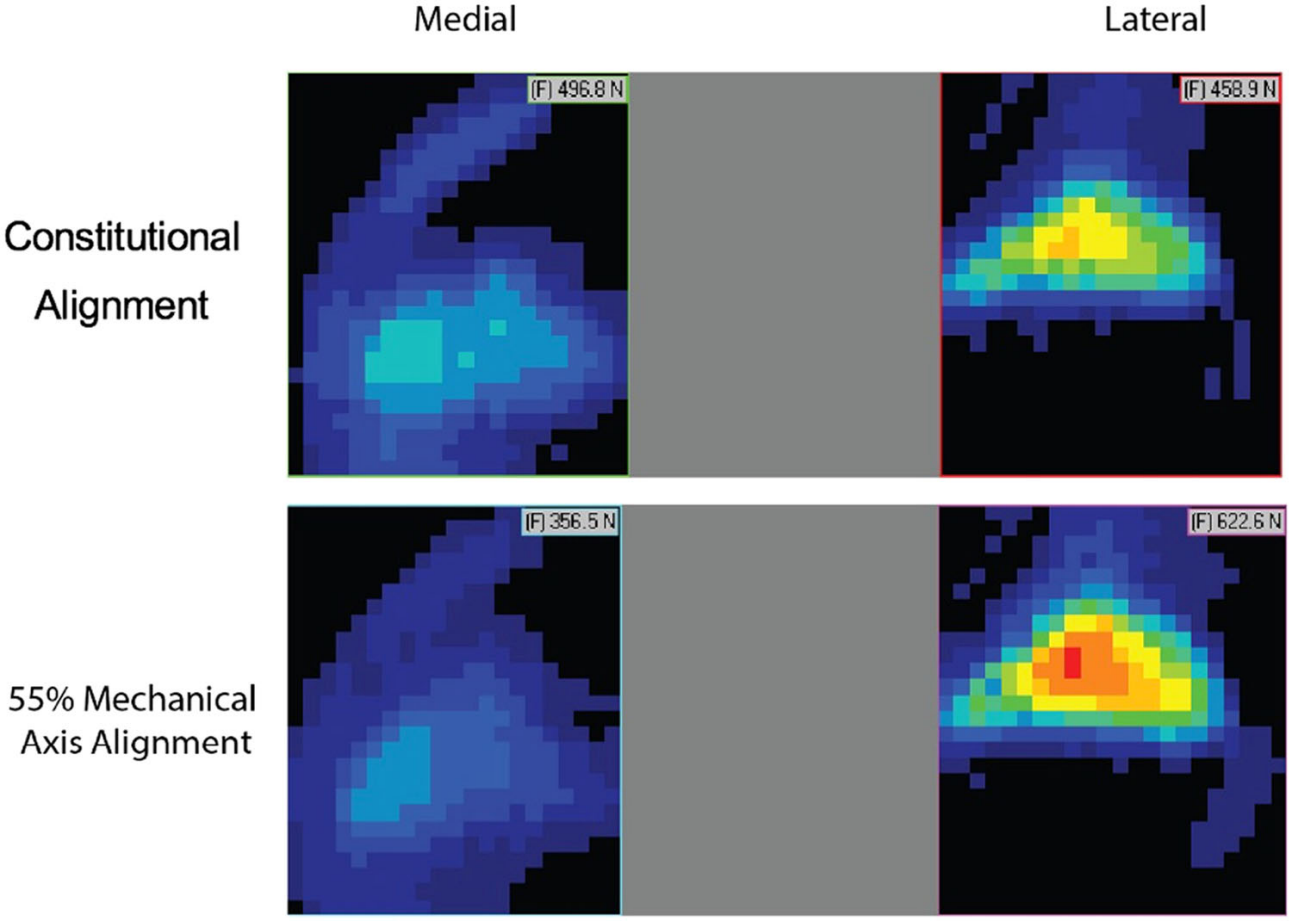
Contact pressure maps of tibiofemoral compartments at constitutional and 55% mechanical axis alignment. Medial contact pressure decreases and lateral contact pressure increases following medial opening‐wedge high tibial osteotomy alignment correction.

With the sMCL intact, altering alignment was found to significantly impact medial and lateral normalised CP (*p* = 0.01; *p* = 0.02). Subsequent pairwise comparisons revealed a statistically significant decrease in medial normalised CP from constitutional alignment to 55% TPW and 60% TPW by 19.0% and 13.9%, respectively (*p* = 0.02; *p* = 0.02) (Figure 7). Additionally, medial normalised CP at 55% TPW was found to be 6.6% lower than at 50% TPW (*p* = 0.046) and 10.4% lower than at 65% TPW (*p* = 0.03). Pairwise comparisons of lateral normalised CP revealed an increase from constitutional alignment to 55% TPW by 14.8% (*p* = 0.017). Additionally, lateral normalised CP at 55% TPW was found to be 6.3% higher than at 50% TPW (*p* = 0.008) and 7.7% higher than at 65% TPW (*p* = 0.044).

### Effects of sMCL release

**Figure 8:**
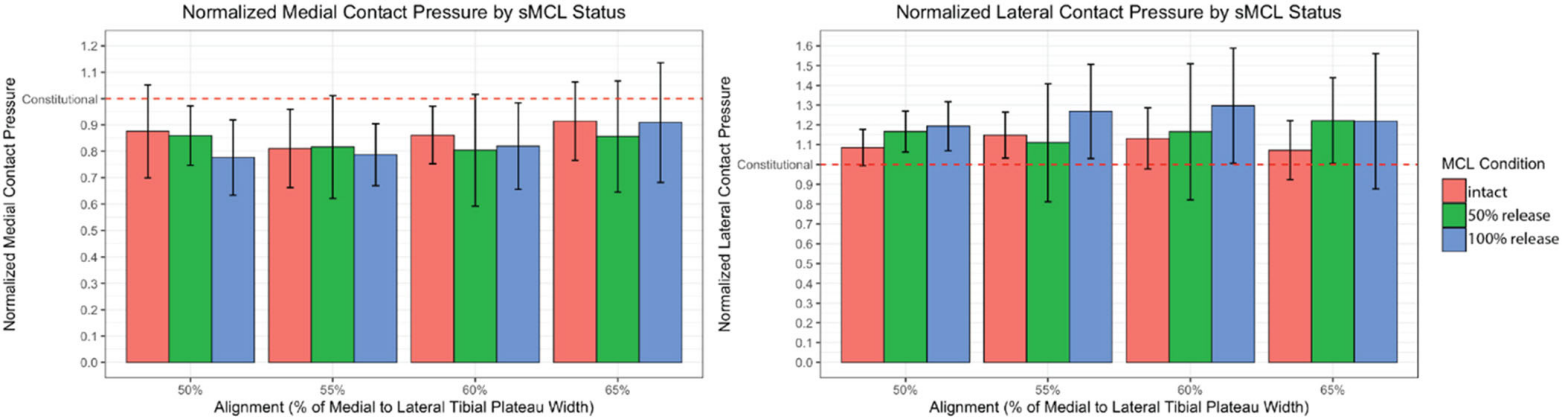
Normalised medial and lateral contact pressure (mean, SD). Pressures are normalised to pressure in the constitutional alignment condition for each superficial medial collateral ligament (sMCL) group. 100% sMCL release lead to significant increases in lateral contact pressure relative to the intact and 50% sMCL release conditions. Across all sMCL conditions, medial contact pressure significantly decreased from constitutional alignment to 50%, 55% and 60% tibial plateau width (TPW) and was lower at 55% TPW than at 65% TPW. Across all sMCL conditions lateral contact pressure significantly increased from constitutional to 50%, 55% and 60% TPW.

In considering the impact of sMCL releases, the interaction between alignment and sMCL release was not found to be significant for the medial and lateral normalised CP (*p* = 0.61; *p* = 0.24) (Figure 8; Table 3). The main effect of sMCL release was found to be statistically significant for lateral compartment normalised CP (*p* < 0.001) but not for medial compartment normalised CP (*p* = 0.24). A subsequent pairwise comparison revealed that the 100% sMCL release condition had 10.8% greater lateral normalised CP than the sMCL intact condition (*p* < 0.001) and 6.3% greater than the 50% sMCL release condition (*p* = 0.026).

**Table 3:** Medial and lateral compartment normalised contact pressure according to alignment and superficial medial collateral ligament (sMCL) condition.

Alignment (% of medial to lateral tibial plateau width [TPW])	Medial contact pressure (CP) (mean ± SD; normalised to constitutional alignment = 1)			Lateral CP (mean ± SD; normalised to constitutional alignment = 1)		
	Intact sMCL	50% sMCL release	100% sMCL release	Intact sMCL	50% sMCL release	100% sMCL release
50%	0.88 ± 0.18	0.86 ± 0.11	0.78 ± 0.14	1.09 ± 0.09	1.17 ± 0.10	1.19 ± 0.12
55%	0.81 ± 0.15	0.82 ± 0.19	0.79 ± 0.12	1.15 ± 0.12	1.11 ± 0.30	1.27 ± 0.24
60%	0.86 ± 0.11	0.80 ± 0.21	0.82 ± 0.16	1.13 ± 0.15	1.17 ± 0.34	1.30 ± 0.29
65%	0.91 ± 0.15	0.86 ± 0.21	0.91 ± 0.23	1.07 ± 0.15	1.22 ± 0.22	1.22 ± 0.34

*Note*: Medial and lateral contact pressure (normalised to constitutional alignment = 1) according to alignment and sMCL condition. Values represent means with standard deviation.

Across all sMCL conditions, alignment was found to have a significant effect on medial and lateral normalised CP (*p* = 0.003; *p* = 0.006) (Figure 8; Table 3). Subsequent pairwise comparisons revealed a statistically significant decrease in medial normalised CP from constitutional alignment to 50% TPW, 55% TPW and 60% TPW by 16.3%, 19.6% and 17.2%, respectively (*p* = 0.01; *p* = 0.004; *p* = 0.01). Additionally, medial normalised CP at 65% TPW was found to be 8.9% greater than at 55% TPW (*p* = 0.03). Pairwise comparisons also revealed statistically significant increases in lateral normalised CP from constitutional alignment to 50% TPW, 55% TPW and 60% TPW by 14.8%, 17.6% and 19.8%, respectively (*p* = 0.008; *p* = 0.004; *p* = 0.047).

## DISCUSSION

The main finding of the present study was that arthritic knees did not differ from healthy knees in their response to MOW‐HTO alignment corrections. From this standpoint, presence of mild‐moderate arthritis may not be a significant factor in influencing the loading biomechanics of cadaver knees undergoing MOW‐HTO. Alignment was a significant factor in decreasing medial compartment pressure with correction to 55% of TPW having the lowest pressure when compared to both constitutional alignment and 65% of TPW. sMCL status did not affect medial compartment pressure but did reveal significant increases in lateral compartment pressure with 100% sMCL release when compared to the intact and 50% sMCL release conditions implying its potential role in overloading the healthy lateral compartment.

Despite good long‐term clinical outcomes of MOW‐HTO, the benefits of surgery from a biomechanical perspective are still not well understood [10, 23]. Many cadaver studies assess medial compartment CP as the primary outcome measure; however, the relationship between decreases in medial CP and reduction in pain and symptoms associated with OA remains unclear. In 2018, Messier et al. showed that intentional weight loss of 10% bodyweight significantly improved pain and functional knee scores in overweight and obese individuals with OA [16]. By normalising the CPs to constitutional alignment, observed decreases in pressure were better interpreted in the context of the findings of Messier et al. The majority of the significant findings exceed what may reflect a clinically meaningful change of 10% and findings less than 10% may need to be interpreted with caution.

Most biomechanical studies investigating MOW‐HTO utilise cadaver knees that lack primary indications of articular degeneration, medial joint space loss and tibial varus alignment deformity [1, 4, 18, 26]. These exclusions may be implemented due to challenges in obtaining appropriate cadaver knee specimens, as well as the difficulty controlling for unintended confounding variables. Tomczyk et al. highlighted the unique morphological characteristics of arthritic knees with varus deformity which may be important to consider in pre‐operative planning of MOW‐HTO [29]. In 1998, Riegger‐Krugh et al tested the biomechanics of lateral closing‐wedge high tibial osteotomy in knees with degenerative joint disease and found that CP was highest along the borders of arthritic lesions [25]. However, the literature has not been clear as to the impact of unique morphological characteristics of healthy versus arthritic knees on loading biomechanics and responses to MOW‐HTO alignment corrections. Due to the lack of significant interaction of arthritic status and alignment in the present study, it was concluded that both healthy are arthritic knees respond similarly to MOW‐HTO alignment corrections from a biomechanical standpoint. Both the healthy and arthritic groups saw a decrease in medial CP between constitutional alignment and 55% TPW and 60% TPW that exceeded the clinically meaningful difference of 10% reduction in relative pressure (Figure 6; Table 2).

The optimal amount of correction in MOW‐HTO is a topic that continues to be debated and has not been thoroughly investigated from a biomechanical perspective. Seitz et al and Ogden et al investigated the effect of 5° and 10° MOW‐HTO corrections on CP and load in the tibiofemoral compartments [18, 26]. Seitz et al. did not find any effect of osteotomy angle on medial CP. Ogden et al. found small differences in medial compartment load after a 5° correction, while a 10° correction led to a decrease of 36.4% ± 24.4%. In contrast, this study found a significant relative decrease in medial CP from constitutional alignment to 50%, 55% and 60% of TPW, whereas there was no difference between constitutional alignment and correction to 65% of TPW across all sMCL conditions (Figure 8, Table 3). As such, the present study suggests that the greatest effect of MOW‐HTO is observed following the initial correction to 50%–60% of TPW, while the biomechanical benefit of correcting to 65% of TPW has diminishing returns in terms of reducing medial compartment CP. Although the present study's findings do not align with those of Seitz et al. and Ogden et al., they do add biomechanical plausibility and support for the clinical trend towards corrections less than the traditional 62% of TPW [8, 12, 18, 26].

The incongruence in the above findings may be due to differences in testing set up and in the method of measuring alignment. The novel testing rig used in the present study aimed to manipulate varus‐valgus angulations in a manner that would maintain a mechanical axis that is parallel to the vertical loading axis of the MTS (Figures 3 and 4). The radius of the convex surfaces upon which the pots of the femur and tibia rotated along were designed to ensure that the centre of the simulated femoral head and centre of the simulated talus would remain fixed in the centre of the curved surface's completed circle (Figures 3 and 4). This ensured that load from the MTS was being transmitted across the knee joint along the same vector as the weight‐bearing axis or mechanical axis of the simulated lower limb. The testing rigs utilised by Seitz et al and Agneskirchner et al manipulate varus‐valgus alignment around a concave arc which may result in a mechanical axis that is oblique to the loading axis of the MTS [1, 26], and therefore could account for differing findings. In contrast, this study's testing scenario aimed to represent the clinical goal of correcting alignment while maintaining a mechanical axis that better simulates the in vivo condition.

The optimal amount of sMCL release in MOW‐HTO to effectively unload the medial compartment continues to be a topic of debate. In 2007, Agneskirchner et al. found that MOW‐HTO correction to 62% of TPW paradoxically led to an increase in medial CP and only found a significant reduction in medial CP following a 100% release of the sMCL [1]. As corrections beyond 55% of TPW are less common in current clinical practice, this study aimed to characterise the effect of sMCL release at corrections to 50%–55% of TPW. Seitz et al. showed that sMCL strain did not significantly differ between constitutional alignment and a relatively small 5° osteotomy condition [26]. This finding was the basis of the hypothesis that a 50% sMCL release may be adequate in unloading medial compartment CP at smaller alignment corrections. This study did not find a significant interaction between alignment and sMCL condition nor a significant effect of sMCL condition on medial CP (Figure 8, Table 3). However, it did find significant relative increases in lateral CP with a 100% sMCL release when compared to the intact and 50% release conditions (Figure 8, Table 3). As such, the findings of this study suggest that while the sMCL release may not be as important for unloading of the medial compartment, it may be important to consider when planning a complete sMCL release while concerned about overloading the healthy lateral compartment.

There are several limitations to the present study. Precisely replicating alignment via the testing apparatus between sMCL conditions was challenging. Ideally, sMCL conditions would be tested sequentially from intact sMCL, partially released sMCL, and completely released sMCL at a fixed alignment to avoid inconsistency in re‐setting alignment. This was the testing design used by Agneskirchner et al. who tested partial and complete sMCL release at a fixed alignment of 62% of TPW [1]. However, unlike Agneskirchner et al.'s study, this study tested the effect of sMCL release across five separate alignments, and thus, were required to re‐adjust alignments within each sMCL condition. Randomisation with respect to sMCL release was not possible as the intact state must be tested before transected states. To account for the variability in setting alignment, pressure values were normalised within each sMCL condition to the pressure at constitutional alignment. This allowed us to assess relative decreases in pressure from constitutional alignment within each sMCL group. It also allowed us to better clinically correlate the findings to the threshold for a clinically meaningful effect of 10% based on the findings of Messier et al. [16].

To further minimise variability, the varus‐valgus alignment was constrained for each condition to ensure that the alignment measured was the alignment being tested during loading. In the in vivo setting, alignment is determined by a complex interplay of bony anatomy and soft tissue structures such as the sMCL which acts as a valgus restraint. Fixing varus‐valgus alignment may have minimised the influence of the sMCL on alignment and reduced the ability of the joint surfaces to optimise contact. Seitz et al. similarly fixed varus and valgus alignment in the sMCL intact and released loading conditions [25]. This is the challenge faced in this type of study design where measurements are to be recorded at fixed alignments. By leaving the remaining degrees of motion unconstrained, the effect of sMCL or joint congruency was not completely negated. However, investigating the effect of sMCL release with alignment unconstrained may be informative, while accepting that alignment may dynamically change during loading.

Additionally, the size of the wedge is intimately related to how much the sMCL is tensioned. For example, a larger tibial varus deformity will require a larger wedge size to achieve a particular mechanical axis alignment, and by extension, a larger wedge size will tension the sMCL to a greater extent. In this study, the wedge was opened as much as was necessary to achieve the goal alignments. The sample of knees fell in a narrow range in terms of tibial varus deformity (Table 1); however, differing wedge sizes is still a potential confounder which could be important to control for or analyse in future studies.

Finally, the biomechanical evaluation was limited to loading in full extension. In reality, pain and symptoms associated with OA occur dynamically throughout activities such as gait and knee flexion (i.e., climbing stairs). Previous studies have investigated the biomechanics of MOW‐HTO in 30° flexion as well as dynamic loading between 10° and 90° flexion [3, 26]. De Pieri et al. investigated redistribution of compartment pressures using kinematic modelling in patients who underwent MOW‐HTO [22]. Investigating pressure changes in flexion and throughout gait would be important to characterise potential orientation and dynamic effects of arthritic status, alignment and sMCL release.

## SUMMARY/CONCLUSIONS

The results of this study characterised the similarities in the response of healthy and arthritic knees to MOW‐HTO alignment corrections. MOW‐HTO correction 55% was shown to be the most effective in decreasing relative medial CP from constitutional alignment. Further, sMCL release did not impact medial compartment CP; however, it did significantly increase relative lateral compartment CP when completely released which may be an important consideration when overcorrection is a concern. Overall, this study elucidates the loading biomechanics of MOW‐HTO across a wide spectrum of clinically relevant alignment corrections and sMCL releases which may help clinicians better understand the biomechanical implications of their unique surgical plans.

## AUTHOR CONTRIBUTIONS

Hassan Syed, Cari Whyne and Sebastian Tomescu designed the study in consultation with David Wasserstein, Naveen Chandrashekar and Aaron Nauth. Hassan Syed, Aadil Mumith, Sebastian Tomescu carried out the experiments. Hassan Syed and Sebastian Tomescu performed the data analysis. Hassan Syed wrote the manuscript. All authors provided edits and revisions of the manuscript and approved it for final submission.

## CONFLICT OF INTEREST STATEMENT

The authors declare no conflicts of interest.

## ETHICS STATEMENT

This study was approved by the Research Ethics Board at Sunnybrook Research Institute REB# 1621. Informed consent was not obtained due to the nature of the study (cadavers).

## Data Availability

The data collected and/or reported is available by request from the corresponding author.
